# 

**Published:** 1882-07-20

**Authors:** 


### TABLE OF CONTENTS.

ORIGINAL AND SELECTED ARTICLES.

Electricity in its Relations to General Practice, by Thomas F. Houston, M.D., of Clarkesville, Ga., 241. The Therapeutics of Tea and Coffee, by F. E. Stewart, M.D., Ph. G., N. Y. City, 245. The Treatment of Carbuncle, by S. Baruch, M.D., N. Y. City, 248. The Treatment of Consumption, by M. L. James, M.D., Richmond, Va, 250. Successful Reduction of a Backward Dislocation of the Radius and Ulna, by George E. Brewer, M. D., of Buffalo, N. Y., 253. Washing of the Stomach, by Austin C. Ramsby, of Chicago, 254. Illness Caused by Filth in Milk, 256. Letter from our Correspondent at the American Medical Association, 257.

ABSTRACTS AND GLEANINGS.

Pathology and Microscopy, 261. Hereditary Alcoholic Appetite, 262. Mercury used in Dentistry, 263. Autopsy upon the Body of Guiteau, 264. Oxyuris Vermicularis, 265. Dr. Byrd on Surgery, 266. Syphilitic Infection of the Finger by Medical Men; Iodoform in Gastric Ulcer, 267. Treatment of Acute Tonsilitis by Ice and Quinine: Poisoning by Winslow's Soothing Syrup; Abortive Treatment of Buboes, 268. Necrosis from Arsenic; Neuralgia, 269. Oral and Dental Surgery; Vaccination after Small Pox; Prescription for Membranous Dysmenorrhœa, 270.

SCIENTIFIC ITEMS.

Demonstration of a New Method, 171. New Theory of the Formation of Coal; Metalic Writing Pencils; Baptiste-Jacob, the New Siamese Twins, 272.

PRACTICAL NOTES AND FORMULÆ.

A Common Error that should be Corrected; Treatment of Uterine Fibroids, 273. Chronic Ulcer; Comedone Wash; New Treatment for Vaginitis; Mercurial Salivation, 274. Camphorated Dover Powders; Treatment of Amenorrhœa; Ring Worm; Chordee, 275.

EDITORIALS AND MISCELLANEOUS.

Editorial Notices; Medical Colleges and Quackery, 276. Officers American Medical Association, 277. Dr. Westmoreland and Atlanta Medical College, 278. The Garfield Doctors' Bills, 279. Special Notices, 280.

